# The polar and lateral flagella from *Plesiomonas shigelloides* are glycosylated with legionaminic acid

**DOI:** 10.3389/fmicb.2015.00649

**Published:** 2015-06-26

**Authors:** Susana Merino, Eleonora Aquilini, Kelly M. Fulton, Susan M. Twine, Juan M. Tomás

**Affiliations:** ^1^Departamento de Microbiología, Facultad de Biología, Universidad de BarcelonaBarcelona, Spain; ^2^National Research Council, OttawaON, Canada

**Keywords:** *Plesiomonas shigelloides*, polar flagella, lateral flagella, *O*-glycosylation, legionaminic acid

## Abstract

*Plesiomonas shigelloides* is the unique member of the *Enterobacteriaceae* family able to produce polar flagella when grow in liquid medium and lateral flagella when grown in solid or semisolid media. In this study on *P. shigelloides* 302-73 strain, we found two different gene clusters, one exclusively for the lateral flagella biosynthesis and the other one containing the biosynthetic polar flagella genes with additional putative glycosylation genes. *P. shigelloides* is the first *Enterobacteriaceae* were a complete lateral flagella cluster leading to a lateral flagella production is described. We also show that both flagella in *P. shigelloides* 302-73 strain are glycosylated by a derivative of legionaminic acid (Leg), which explains the presence of Leg pathway genes between the two polar flagella regions in their biosynthetic gene cluster. It is the first bacterium reported with *O*-glycosylated Leg in both polar and lateral flagella. The flagella *O*-glycosylation is essential for bacterial flagella formation, either polar or lateral, because gene mutants on the biosynthesis of Leg are non-flagellated. Furthermore, the presence of the lateral flagella cluster and Leg *O*-flagella glycosylation genes are widely spread features among the *P. shigelloides* strains tested.

## Introduction

*Plesiomonas shigelloides* is a Gram-negative bacilli flagellated bacterium. This facultative anaerobic bacterium is ubiquitous, has been isolated from different water sources (freshwater or surface water), and animals (wild and domestic; Farmer et al., 1992). In humans, *P. shigelloides* is associated with diarrheal disease in humans (Brenden et al., 1988). Sometimes could also be the cause of gastroenteritis, including acute secretory gastroenteritis (Mandal et al., 1982), an invasive shigellosis-like disease (McNeeley et al., 1984), and a cholera-like illness (Tsukamoto et al., 1978). Extra intestinal infections, such as meningitis, bacteremia (Billiet et al., 1989), and pseudoappendicitis (Fischer et al., 1988), are also associated with *P. shigelloides* infection. Of particular concern are the severe cases of meningitis and bacteremia (Fujita et al., 1994) caused by *P. shigelloides*.

*Plesiomonas shigelloides* was initially classified in the *Vibrionaceae* family; however, molecular studies by Martinez-Murcia et al. (1992) indicated that is related to the enterobacterial genus *Proteus* phylogenetically. Huys and Sings (1999) during studies of *Aeromonas* spp. genotyping using by the amplified fragment length polymorphism found that *P. shigelloides* clearly falls out of the major *Aeromonas* cluster. According to these features the genus *Plesiomonas* was reclassified to the family *Enterobacteriaceae*, being the only oxidase-positive member of this family (Garrity et al., 2001). In order to distinguishing different strains of *P. shigelloides*, two major serotyping schemes, one based on *O*-antigen lipopolysaccharide (O) and the other one on flagellar (H) antigens. With a total of 102 somatic antigens and 51 flagellar antigens recognized (Aldova and Shimada, 2000).

The flagella biosynthesis, in terms of resources and energy, is a costly commitment for the bacterium (Macnab, 1996). The flagella number is variable, and the distribution most frequently found on pathogenic bacteria are monotrichous (single flagellum) or pertitrichous (multiple flagella around the cell; Macnab, 1996). The flagella expression is dependable of the growth conditions. When grown in plates, several bacterial species produced more flagella than when they grow in liquid medium. Some species, like *Proteus mirabilis*, have been observed to show an increase in the numbers of flagella. *Vibrio parahaemolyticus*, have a single polar flagellum in liquid medium, instead when grown on solid medium, produces the polar flagellum (Fla) and peritrichous (or lateral) flagella (Laf; Allison and Hughes, 1991; Allison et al., 1992; Merino et al., 2014). Lateral flagella, were shown in about seven other *Vibrio* species (some of which evokes a disease spectrum similar to *V. parahaemolyticus*; Shinoda et al., 1992), while only a reduced number of bacterial species, including *Rhodospirillum centenum* (a purple photosynthetic bacterium; McClain et al., 2002), *Azospirillum* spp. (nitrogen-fixing rhizobacteria that colonize plants; Moens et al., 1996), *Helicobacter mustelae* (the causative agent of chronic gastritis and ulcer disease in ferrets; O’Rourke et al., 1992), *P. shigelloides* (Inoue et al., 1991), and *Aeromonas* spp. (opportunistic and gastroenteric pathogens of man; Gavín et al., 2002). Other species that show lateral flagella include *Bradyrhizobium japonicum* (Kanbe et al., 2007), *Photobacterium profundum* (Eloe et al., 2008), and *Rhodobacter sphaeroides* (Poggio et al., 2007). Furthermore, *Selenomonas ruminantium* subsp. *lactilytica* is a solely laterally flagellate bacterium (Haya et al., 2011).

Protein glycosylation is one of the most common protein post-translational modifications and consists in the covalent attachment of carbohydrates to amino acids. This mechanism was thought to occur exclusively in eukaryotes. However, protein glycosylation systems have been identified in all forms of life including prokaryotes. *N*-glycosylation is the covalent linkage to asparagine residues of carbohydrates, while *O-*glycosylation to serine or threonine residues. *O-*glycosylation in bacteria has been largely reviewed recently (Iwashkiw et al., 2013). As more bacterial genomes are now available together with bioinformatic analysis coupled with functional analysis, the elucidation of glycosylation pathways achieved increasing, including the identification of many genes that participate in flagellin glycosylation (Iwashkiw et al., 2013). The number of *O-*glycosylation genes involved is diverse in each bacterial species (Goon et al., 2003; Schirm et al., 2003; Faridmoayer et al., 2007; Iwashkiw et al., 2012). In spite of these advances, the knowledge of glycans structure and composition of which modify from Gram-negative bacteria flagellins is restricted to certain species and has been observed to be strain-dependent [as reviewed by Merino and Tomás (2014)].

In this work we study the genetics of *P. shigelloides* flagella (polar and lateral), and their flagella post-translational modifications, the first report of flagellar glycosylation in enteric bacteria.

## Materials and Methods

### Bacterial Strains, their Growth Conditions, and Plasmids Used

The bacterial strains, as well as the plasmids used, are listed on **Table 1**. Bacteria were grown in TSB broth and TSA medium supplemented if necessary with kanamycin (25 μg/ml), tetracycline (20 μg/ml), and rifampicin (100 μg/ml) when needed.

**Table 1:** Bacterial strains and plasmids used.

Strain or plasmid	Relevant characteristics^a/^	Source or reference
***Escherichia coli***		
DH5α	F- *endA hsdR17* (rk^-^ mk^+^) *supE44 thi-1 recA1 gyr-A96 80lacZ*	Hanahan (1983)
S17-1λpirKm1	*thi thr1 leu tonA lacY supE recA*::*RP4-2* (*Tc*::*Mu*)*Km^r^* λ*pir* with miniTn5Km1	De Lorenzo et al. (1990)
MC1061λpir	*thi thr1 leu6 proA2 his4 argE2 lacY1 galK2 ara14 xyl5 supE44* λ *pir*	Rubirés et al. (1997)
***Plesiomonas Shigelloides***		
302-73	Wild type, serotype O12:K80	Pieretti et al. (2010)
302-73R	302-73, spontaneous Rif^r^	Aquilini et al. (2013)
A	302-73*flgE*:mini-Tn5Km1 Rif^r^ Km^r^	This study
B	302-73*flhA*:mini-Tn5Km1 Rif^r^ Km^r^	This study
C	302-73*fliI*:mini-Tn5Km1 Rif^r^ Km^r^	This study
D	302-73*flgK*:mini-Tn5Km1 Rif^r^ Km^r^	This study
E	302-73*lafA*:mini-Tn5Km1 Rif^r^ Km^r^	This study
F	302-73*flhA*_L_:mini-Tn5Km1 Rif^r^ Km^r^	This study
G	302-73*flgE*_L_:mini-Tn5Km1 Rif^r^ Km^r^	This study
H	302-73*ptmA*:mini-Tn5Km1 Rif^r^ Km^r^	This study
I	302-73*legH*:mini-Tn5Km1 Rif^r^ Km^r^	This study
**Plasmids**		
pLA2917	Tc^r^, Km^r^	Allen and Hanson (1985)
COS-FLAregI-1	pLA2917 with 20-kb chromosomal 302-73 *Sau*3A insert carrying part of the polar flagella biosynthesis region I,Tc^r^	This study
COS-LAFI	pLA2917 with 20-kb chromosomal 302-73 *Sau*3A insert carrying part of the lateral flagella biosynthesis region, Tc^r^	This study
COS-LEG	pLA2917 with 20-kb chromosomal 302-73 *Sau*3A insert carrying complete Leg biosynthesis region, Tc^r^	This study
pRK2073	Helper plasmid, Sp^r^	Canals et al. (2006)
pGEM-T	PCR cloning vector, Amp^r^	Promega
pDM4	*pir* dependent with *sacAB* genes, oriR6K, Cm^R^	Milton et al. (1996)
pDM4Δ*pgmL*	pDM4 with truncated in frame *pgmL*	This study
pDM4Δ*legF*	pDM4 with truncated in frame *legF*	This study
pBAD33	Arabinose inducible expression vector, Cm^R^	ATCC
pBAD33-*pgmL*	pBAD33 with complete *pgmL*	This study
pBAD33-*legF*	pBAD33 with complete *legF*	This study

^a/^ = *resistant*.

### MiniTn5Km-1 Mutagenesis

Conjugal transfer of miniTn5Km-1 transposition element from *Escherichia coli* S17-1λ*pir*Km-1 to *P. shigelloides* 302-73R (wild type strain rifampicin-resistant) was carried out in a conjugal drop as previously described (Aquilini et al., 2013).

### Construction of a *P. shigelloides* Genomic Library

*Plesiomonas shigelloides* strain 302-73 (serotype O1) genomic DNA was isolated and partially digested with *Sau3A* as described by Sambrook et al. (1989). The *P. shigelloides* strain 302-73 genomic library, using cosmid pLA2917 (Allen and Hanson, 1985), was performed as described (Guasch et al., 1996).

### General DNA Methods

General DNA manipulations were done essentially as previously described described (Sambrook et al., 1989; Aquilini et al., 2014).

### Southern Blot Hybridizations

Southern blotting was performed by capillary transfer (Sambrook et al., 1989) from the gel to a nylon membrane (Hybond N1, Amersham). Probe labeling, hybridization, and detection were carried out as previously described (Aquilini et al., 2014) using the enhanced chemiluminescence labeling and detection system (Amersham) according to the manufacturer’s instructions.

### DNA Sequencing and *In Silico* Analysis of Sequence Data

These studies were previously described (Wilhelms et al., 2013). The dideoxy-chain termination method (Sanger et al., 1977), BLAST (Altschul et al., 1997; Bateman et al., 2002), and Clustal W were used.

### Complementation Studies

Complementation of the different mutants carrying the miniTn5 was done as previously described (Aquilini et al., 2013) by conjugal transfer of positive recombinant clones from the genomic library.

### Antisera

Anti-*P. shigelloides* polar flagellum and lateral flagella serum were independently obtained using purified polar flagellum or lateral flagella obtained after cesium chloride, and assayed as previously described for other surface molecules (Tomás et al., 1991; Merino et al., 1992).

### Motility Assays (Swarming and Swimming)

The studies were performed as previously described (Wilhelms et al., 2012). Bacterial colonies were picked with a sterile toothpick and deposited into the center of swarm agar or swim agar plate. The plates were incubated up for 16–24 h at 25°C and motility was examined by the migration of bacteria through the agar from the center toward the plate periphery. Swimming motility in liquid medium was observed by phase-contrast microscopy at a magnification of x 400 as previously (Wilhelms et al., 2012).

### Transmission Electron Microscopy (TEM)

Transmission electron microscopy (TEM) studies were performed as previously described (Wilhelms et al., 2012).

### Flagella Purification

*Plesiomonas shigelloides* strain 302-73 was grown in TSB for the polar flagellum purification. For the isolation of lateral flagella the strains were grown on TSA and recovered with 100 mM Tris (pH = 7.8). Purified flagella were isolated as previously described (Merino et al., 2014).

### Cytoplasmic Fraction

*Plesiomonas shigelloides* cytoplasmic fraction from strain 302-73 cells grown in TSB at 37°C was obtained as previously described (Wilhelms et al., 2012).

### Immunological Methods

Western blot of cytoplasmic fractions or purified flagella was performed as previously described (Wilhelms et al., 2012). Immunoblotting was carried out as described (Towbin and Gordon, 1984) using specific anti-polar or lateral flagellins polyclonal serum (Canals et al., 2006; 1:2000).

### Electrospray Liquid Chromatography Mass Spectrometry

Mass spectrometry studies of intact flagellin proteins were carried out using 1 μg or less of protein, as described in our previous work (Wilhelms et al., 2012). Briefly, purified flagellin samples were injected onto a protein microtrap (Michrom Bioresources Inc., Auburn, CA, USA) connected to a gradient HPLC pump (Agilent 1100 HPLC). To resolve the proteins, a gradient of 5–60% solvent B (1 mL/min) over 60 min was used, where Solvent A was 0.1% formic acid in HPLC grade water and solvent B was 0.1% formic acid in acetonitrile. A precolumn splitter was used to direct ∼35 μl/min of the HPLC mobile phase through the trap or column and into the electrospray interface of the QTOF2 (Waters, Milford, MA, USA) or Orbitrap XL Mass Spectrometer (Thermal, CA, USA) to allow real-time monitoring of ion elution profiles. Intact masses of proteins were calculated using MaxEnt (Waters, Beverly, MA, USA) software by spectral deconvolution.

To identify potential glycopeptides, flagellin (50–200 μg) was digested and analyzed as previously described (Wilhelms et al., 2012). Unmodified peptides were identified using MASCOT (Matrix Science, London, UK) as described (Wilhelms et al., 2012). Glycopeptide MS/MS spectra were *de novo* sequenced as previously described (Wilhelms et al., 2012).

### Construction of Defined in Frame Legionaminic Acid Mutants and their Complementation

The chromosomal in-frame *pgmL* and *legF* deletion mutants, 302Δ*pgmL* and 302Δ*legF*, respectively, were constructed by allelic exchange as described (Milton et al., 1996), and used by us (Merino et al., 2014). The primers used to obtain the mutants are listed in **Table 2**. Two DNA fragments (A–B and C–D) were obtained after asymmetric polymerase chain reactions (PCRs), then were annealed at their overlapping region, and a single DNA fragment obtained after PCR using primers A and D. pDM4Δ*pgmL* and pDM4Δ*legF* plasmids were obtained as previously described (Merino et al., 2014). These plasmids were transferred by triparental matings using the *E. coli* MC1061 *(λpir*), the mobilizing strain *E. coli* HB101/pRK2073 and *P. shigelloides* mutant 302-73R as recipient strain. Colonies grown on plates with chloramphenicol and rifampicin, were confirmed for genome integration of vector by PCR analysis. Colonies grown rifampicin resistant (Rif^R^) and chloramphenicol sensitive (Cm^S^) after sucrose treatment, PCR confirmed for mutation were chosen.

**Table 2:** **(A)** Primers used in the construction of chromosomal in-frame deletion mutants. **(B)** Primers used for mutant complementation using vector pBAD33.

A	
Primers^a,b^	Amplified fragment
*pgmL*
A: 5′-CGCGGATCCGAACGCTTGAGTCGTGAGT-3′	AB (687 bp)
B: 5′-*TGTTTAAGTTTAGTGGATGGG*ACCCAGCTTCAACACAAAG-3′
C: 5′-*CCCATCCACTAAACTTAAACA*GAAGGCGAAGATCTGGAG-3′	CD (695 bp)
D: 5′-CGCGGATCCTACCAATTCCACCACCAC-3′	
	AD (1403 bp)
*legF*	
A: 5′-GAAGATCTTGCCGTTGGCTACTGTC-3′	AB (684 bp)
B: 5′-*TGTTTAAGTTTAGTGGATGGGA*CCCCGAGCAAATATAAACG-3′	
C: 5′-*CCCATCCACTAAACTTAAACA*AGTCCCAAAGTCACGTTCTG-3′	CD (685 bp)
D: 5′-GAAGATCTATATGCCACCAGGGCTAAC-3′	
	AD (1390 bp)

^a^*Italic letters show overlapping regions.*
^b^*Underlined letters show BamHI or BglII restriction site.*

**B**

**Plasmid**	**Primer**

pBAD33-*pgmL*^a^	PgmL-FOR: 5′-TCCCCCGGGTACACGATGTGCAAG-3′
	PgmL-REV: 5′-GCTCTAGACCACAACCTGCTGTGAC-3′
pBAD33-*legF*^b^	LegF-FOR: 5′-TCCCCCGGGCCTGAGTGGGACAAAAAT-3′
	LegF-REV: 5′-GCTCTAGATCAATGTCAGCAGCAACG-3′

^a^*Primers contain SmaI(bold) and XbaI(underlined), the PCR amplified product (1496 bp) was ligated to SmaI- XbaI digested pBAD33*.

^b^*Primers contain SmaI(bold) and XbaI(underlined), the PCR amplified product (982 bp) was ligated to SmaI- XbaI digested pBAD33*.

Plasmids pBAD33-*pgmL* and pBAD33-*legF* were constructed carrying the wild type genes *pgmL* and *legF* by PCR amplification of genomic DNA by using specific primer pairs and ligated to the plasmid pBAD33 from ATCC (American Type Culture Collection; see the list of primers in **Table 2**). Plasmids pBAD33-*pgmL* and pBAD33-*legF* were introduced in *E. coli* DH5α by electroporation, and then by triparental matings were introduced in the corresponding mutants. Induction or repression of genes in pBAD33 was achieved as described in ATCC.

## Results

**FIGURE 1:**
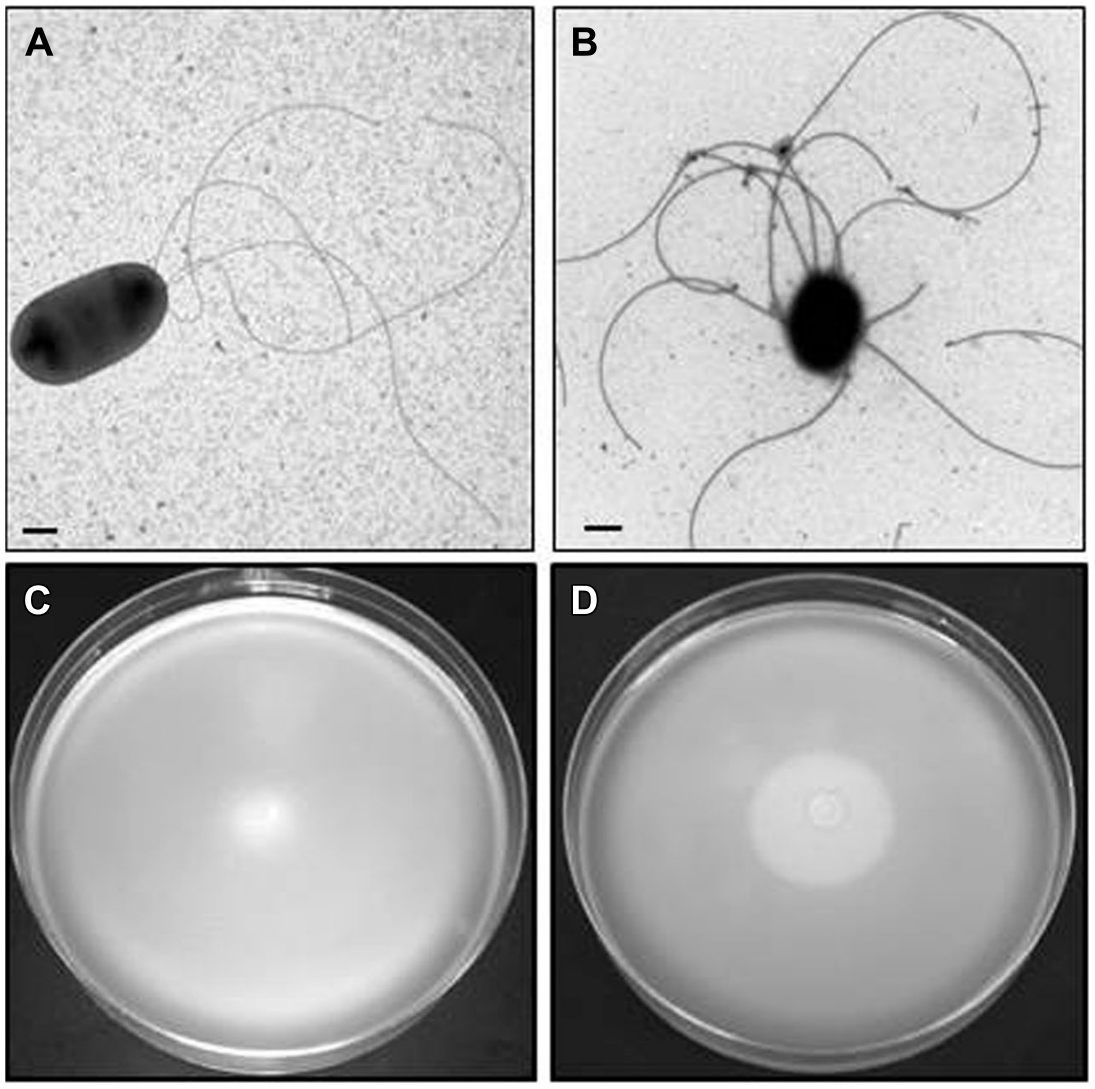
***Plesiomonas shigelloides* 302-73 wild type strain serotype O1.** TEM from cells grown in liquid medium **(A)** and swarming agar plates **(B)**. Motility in swimming **(C)** and swarming **(D)** agar plates.

*Plesiomonas shigelloides* 302-73 [serogroup O1 (Pieretti et al., 2010)] grown in liquid medium or semisolid medium (swimming agar plates) showed the typical three-four flagella located in single point of one cell pole (lophotricus; **Figure 1**). However, when the agar concentration was increased, the flagellar distribution shifted from single pole to more disperse. The agar concentration seems to be involved in this change in flagella distribution. When the bacteria were grown in solid or semisolid media (swarming agar plates), a complete different flagella distribution was observed. As can be seen in **Figure 1** the flagella showed a typical peritrichous distribution over the entire cell surface.

A similar pattern of flagellar distribution with changes in growth medium was observed with 12 *P. shigelloides* strains. Among these strains eight represented five different serotypes (O1, O2, O3, O17, and O54) while four were non-serotyped strains. The source of the strains was from clinical stools (7) and fish (5), from Japan four of them, four from Spain, three from Brazil, and one from Poland.

### MiniTn5Km-1 Mutagenesis

A spontaneous rifampicin-resistant *P. shigelloides* mutant (named 302-73R) derived from the wild type strain 302-73 was isolated by our group. *P. shigelloides* 302-73R showed identical pattern of flagella production as described previously for wild type strain. We selected insertional mutants, as described in Materials and Methods, and grouped by their inability to swim, to swarm, or both negative characteristics.

**FIGURE 2:**
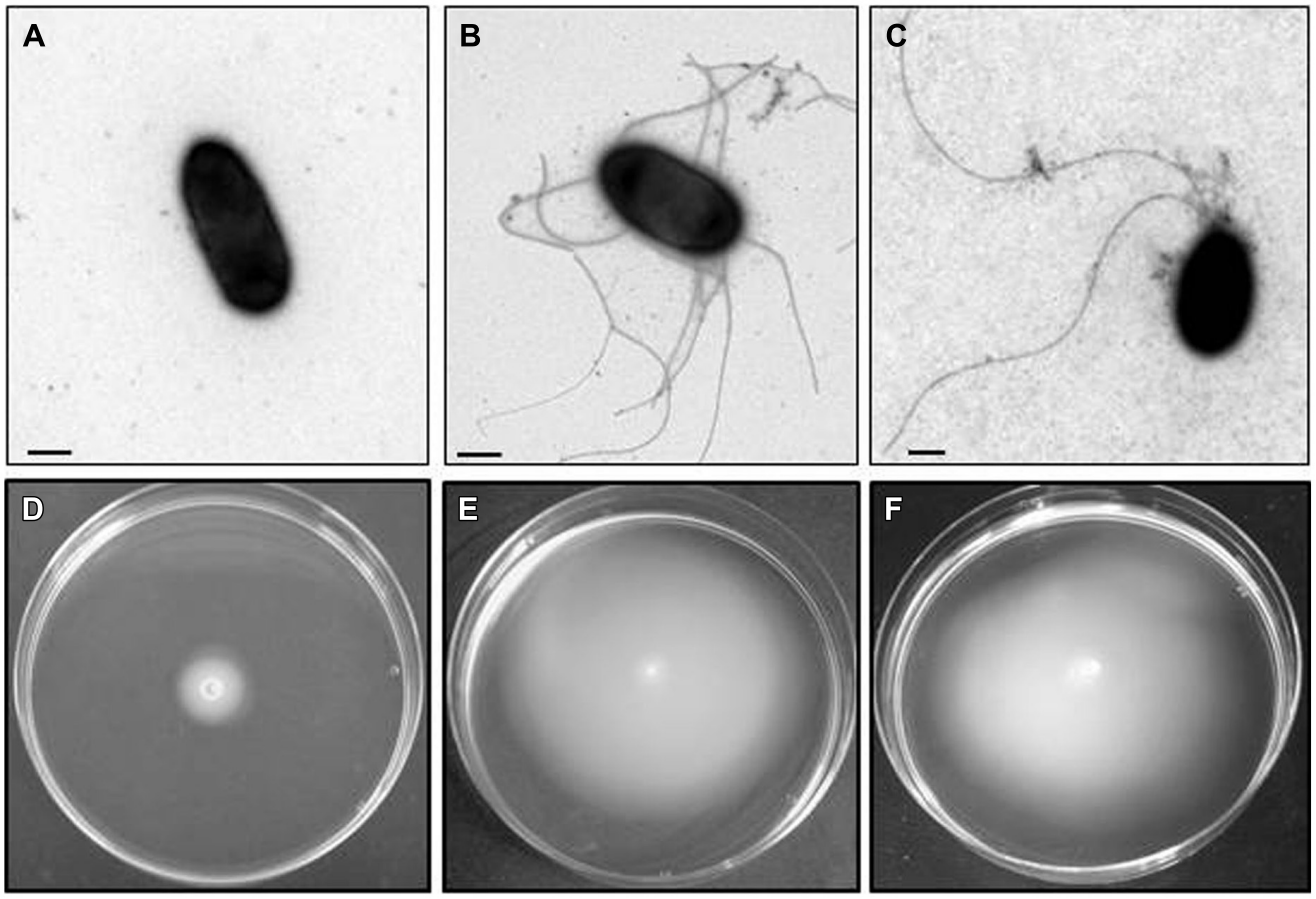
***Plesiomonas shigelloides* A mutant (as an example for the insertional polar flagella mutants).** TEM of the A mutant grown in liquid medium **(A)** and swarming agar plates **(B)** and complemented mutant with COS-FLAregI-1harboring the corresponding wild type gene grown in liquid medium **(C)**. Bar, correspond to 0.5 μm. Motility of the A mutant in swimming **(D)** and swarming **(E)** agar plates. The complemented mutant with COS-FLAregI-1harbouring the corresponding wild type gene in swimming agar plate **(F)**.

**FIGURE 3:**
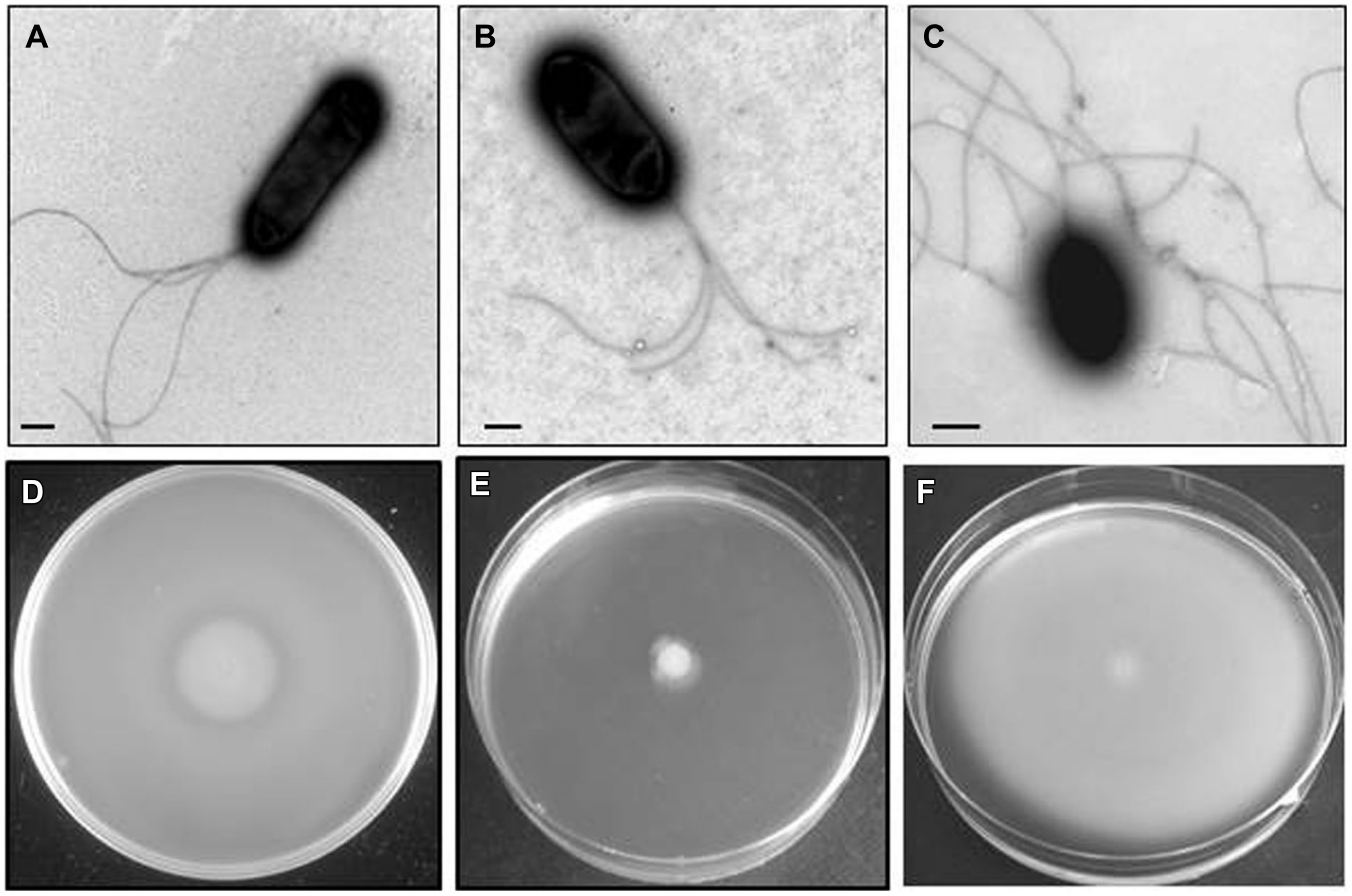
***Plesiomonas shigelloides* E mutant (as an example for the insertional lateral flagella mutants).** TEM of the E mutant grown in liquid medium **(A)** and swarming agar plates **(B)** and complemented mutant with COS-LAFI harboring the corresponding wild type gene grown in semisolid medium **(C)**. As could be observed in B the polar flagella are constitutively expressed in semisolid medium. Bar, correspond to 0.5 μm. Motility of the E mutant in swimming **(D)** and swarming **(E)** agar plates. The complemented mutant with COS-FLAregI-1harbouring the corresponding wild type gene in swarming agar plate **(F)**.

**FIGURE 4:**
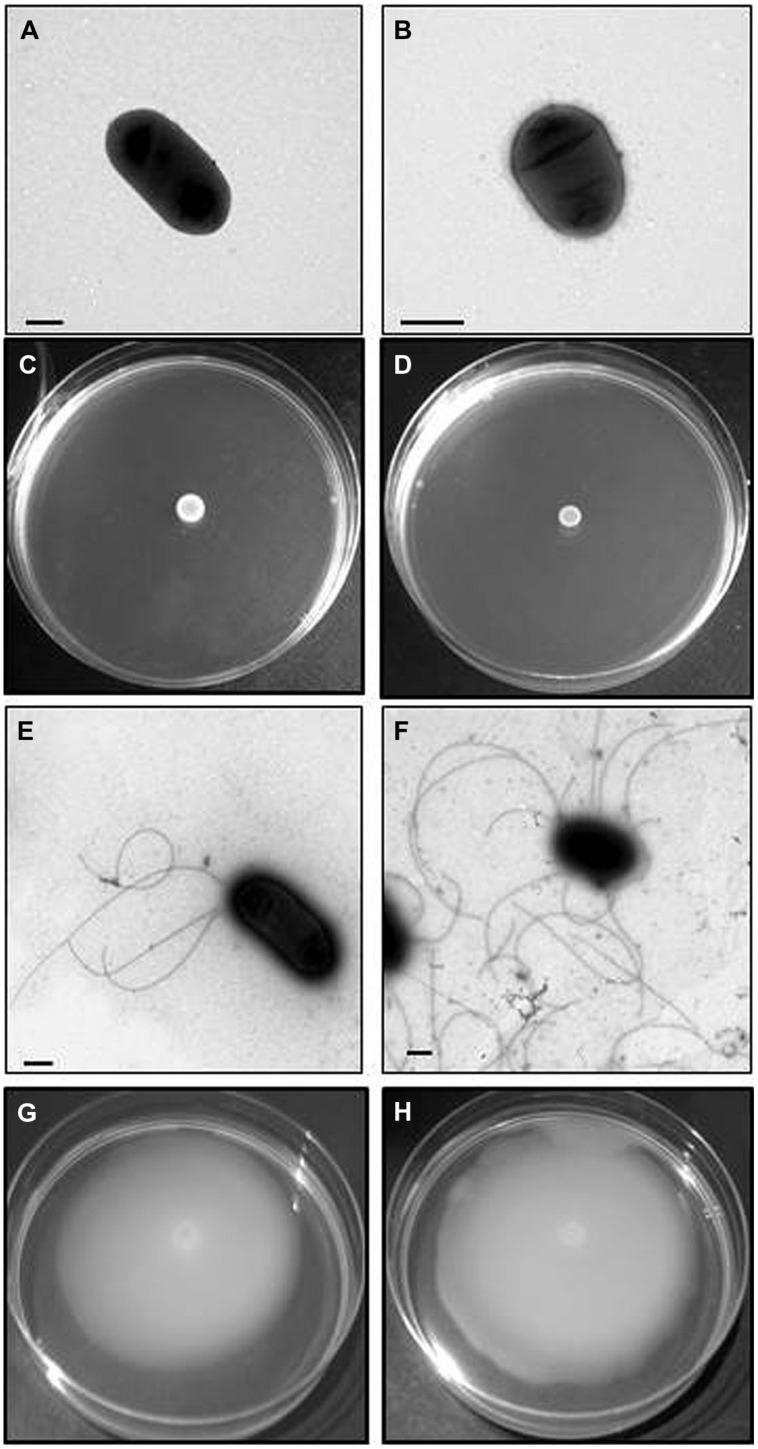
***Plesiomonas shigelloides* H mutant (as an example for the insertional Leg biosynthetic cluster mutants).** TEM of the H mutant grown in liquid medium **(A)** and swarming agar plates **(B)** and complemented mutant with COS-LAFI harboring the corresponding wild type gene grown in liquid **(E)** and semisolid medium **(F)**. Bar, correspond to 0.5 μm. Motility of the H mutant in swimming **(C)** and swarming **(D)** agar plates. The complemented mutant with COS-LEG harboring the corresponding wild type gene in swimming **(G)** and swarming **(H)** agar plates.

Among an initial screening of 2500 colonies four mutants were selected (initially named A, B, C, and D), based upon inability to swim but retaining the ability to swarm. A further, three mutants (initially named E, F, and G) were selected based upon inability to swarm but retaining ability to swim. Lastly, two mutants (initially named H and I) were selected that were unable to swim or swarm. Mutants A, B, C, and D, when observed by EM in appropriate conditions showed lateral flagella but not polar (**Figure 2**), while mutants E, F, and G (**Figure 3**), showed polar but not lateral flagella by EM when grown in appropriate conditions. Mutants H and I were unable to produce polar or lateral flagella observed by EM in any growth conditions (**Figure 4**). The presence of a single copy of the minitransposon in their genome was determined by Southern blot analysis. We were unable to clone the minitransposon-containing DNA fragment from the mutants using methodologies that were successful in other bacteria (Aquilini et al., 2013).

Complementation of the mutants, using a cosmid based genomic library of *P. shigelloides* 302-73 (see Materials and Methods) reversed the phenotype observed, either to swim or swarm in motility plates.

### Polar Flagella Mutants

We found several recombinant positive clones able to complement A, B, C, and D mutants. The complementation was studied by the recovery of swimming behavior under appropriate conditions. All complemented mutants were able to produce polar flagella when observed by EM growing in liquid conditions (**Figure 2**). Sequencing the recombinant positive clones complete inserts revealed the complete region to correspond to PLESHI_03205 to PLESHI_03505 in the complete *P. shigelloides* 302-73 genome (Piqué et al., 2013).

**FIGURE 5:**
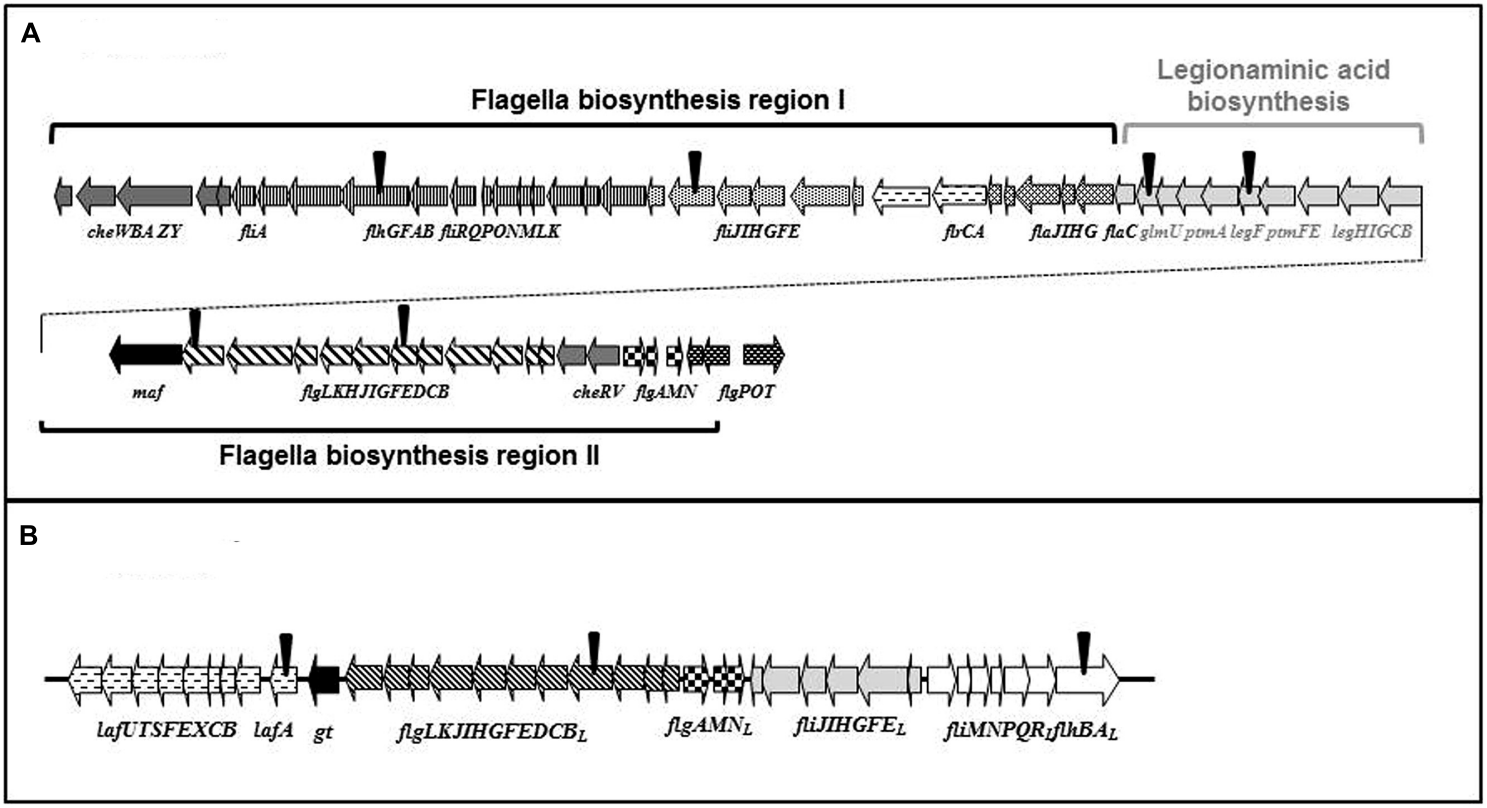
***Plesiomonas shigelloides* 302-73 wild type strain serotype O1 polar **(A)** and lateral **(B)** flagella clusters.** The polar flagella cluster shows two different regions adjacent to Leg biosynthetic genes. The inverted triangles (▼) indicate the position of the different insertional mutants obtained by miniTn5 mutagenesis.

Polar flagella gene cluster, as shown in **Figure 5A**, are based in two gene regions (I and II) adjacent to a group of putative biosynthetic Leg genes. In region I there are several genes encoding chemotaxis proteins, including the σ^28^ factor *fliA*, cluster from *flhB* to *G*, *fliK* to *R*, *fliE* to *J*, *flrA* and *C*, and *flaC* to *J* (transcribed in the same direction). This region I, similar to *V. parahaemolyticus* region two by gene distribution and transcription sense, also lacks the motor genes (McCarter, 2001). Region II, downstream of the putative biosynthetic Leg genes group, contains cluster *flgP*,*O*,*T*, or *flgA*,*M*,*N* with the typical transcription sense in the different Gram-negative bacteria described, two genes encoding chemotaxis proteins, and cluster *flgB* to *L*. By gene distribution and transcription sense this region II is similar to region 1 of *V. parahaemolyticus* and *Aeromonas hydrophila* (McCarter, 2001; Canals et al., 2006).

**Table 3** shows the ORFs with their predicted function based on their homology to proteins of known function. Proteins of unknown function were not included. The last gene in this region encoded an ORF (named Gt), which showed homology to domains of a glycosyltransferase. This was provisionally assigned to the polar flagella cluster and not to the putative biosynthetic Leg genes. Once the DNA fragment was completely sequenced, several primers were used to derive the DNA sequence to locate the miniTn5 [A = *flgE*, B = *flhA*, C = *fliI*, and D = *flgK* (**Figure 5A**)].

**Table 3:** Characteristics of the *P. shigelloides* 302-73 strain polar flagella gene regions I and II.

ORF	Protein name	Protein Size	Predicted function	Homologous protein with known function	Percentage identity/similarity
**Polar flagella region I**
1	CheW	162	Chemotaxis protein	CheW (VP2225) of *Vibrio parahaemolyticus*	84/88
2	CheB	377	Chemotaxis protein	CheB-2 (AHA_1386) of *Aeromonas hydrophila* ATCC7966	72/76
3	CheA	728	Chemotaxis protein	CheA (VP2229) of *Vibrio parahaemolyticus*	66/69
4	CheZ	241	Chemotaxis protein	CheZ (ASA_1356) of *Aeromonas salmonicida* A449 CheZ (VP2230) of *Vibrio parahaemolyticus*	51/59 48/54
5	CheY	127	Chemotaxis protein	CheY (AHA_1383) of *Aeromonas hydrophila* ATCC7966	91/93
6	FliA	240	σ^28^	FliA of *Aeromonas hydrophila* AH-3	67/71
7	FlhG	296	Flagella number regulator	FlhG of *Vibrio alginolyticus*	72/78
8	FlhF	527	Polar flagella site determinant	FlhF (VP2234) of *Vibrio parahaemolyticus*	66/73
9	FlhA	698	Export/assembly	FlhA (VP2235) of *Vibrio parahaemolyticus*	78/81
10	FlhB	377	Export/assembly	FlhB (VP2236) of *Vibrio parahaemolyticus*	60/66
11	FliR	264	Export/assembly	FliR (VP2237) of *Vibrio parahaemolyticus*	51/61
12	FliQ	89	Export/assembly	FliQ (VP2238) of *Vibrio parahaemolyticus*	67/78
13	FliP	261	Export/assembly	FliP (VP2239) of *Vibrio parahaemolyticus*	76/82
14	FliO	139	Export/assembly	FliO of *Vibrio cholerae*	39/41
15	FliN	128	Motor switch	FliN (AHA_1373) of *Aeromonas hydrophila* ATCC7966	72/78
16	FliM	347	Motor switch	FliM of *Aeromonas hydrophila* AH-3	80/87
17	FliL	164	Flagella protein	FliL (AHA_1371) of *Aeromonas hydrophila* ATCC7966	42/46
18	FliK	487	Hook length	FliK of *Vibrio cholerae*	57/68
19	FliJ	146	Export/assembly	FliJ (VP2245) of *Vibrio parahaemolyticus*	43/51
20	FliI	439	Export ATP synthase	FliI (VP2246) of *Vibrio parahaemolyticus*	78/82
21	FliH	322	Export/assembly	FliH (VP2247) of *Vibrio parahaemolyticus*	37/46
22	FliG	342	Motor switch	FliG (AHA_1366) of *Aeromonas hydrophila* ATCC7966	75/82
23	FliF	569	M-ring	FliF (VP2249) of *Vibrio parahaemolyticus*	53/61
24	FliE	107	MS ring/rod adapter	FliE (VP2250) of *Vibrio parahaemolyticus*	58/62
25	FlrC	558	σ^54^-dependent two-components response regulator	FlaM (VP2251) of *Vibrio parahaemolyticus*	61/65
26	FlrA	509	σ^54^-dependent flagella regulator	FlrA of *Vibrio cholerae*	58/65
27	FlaJ	134	Chaperone	FlaJ (VP2254) of *Vibrio parahaemolyticus*	63/69
28	FlaI	94	Flagella rod protein	FlaI (VP2255) of *Vibrio parahaemolyticus*	38/50
29	FlaH	446	Hook-associated protein-2	FlaH (VP2256) of *Vibrio parahaemolyticus*	36/45
30	FlaG	132	Filament length control	FlaG of *Vibrio alginolyticus*	36/41
31	FlaC	377	Flagellin	FlaC (VP0788) of *Vibrio parahaemolyticus*	51/55
**Polar flagella region II**					
1	Gt	691	Glicosyltransferase	BRAO375_790043 of *Bradyrhizobium* sp. AZOBR_p1140113 of *Azospirillum brasilense*	36/42 32/40
2	FlgL	417	Hook-associated protein 3	FlgL of *Vibrio cholerae*	34/42
3	FlgK	639	Hook-associated protein 1	FlgK (VP0785) of *Vibrio parahaemolyticus*	35/41
4	FlgH	230	L-ring	FlgH of *Vibrio cholerae*	56/63
5	FlgJ	322	Peptidoglycan hydrolase	FlgJ (VP0784) of *Vibrio parahaemolyticus*	58/66
6	FlgI	355	P-ring	FlgI (VP0783) of *Vibrio parahaemolyticus*	73/81
7	FlgG	262	Rod	FlgG of *Vibrio cholerae*	69/76
8	FlgF	248	Rod	FlgF (AHA_2838) of *Aeromonas hydrophila* ATCC7966	56/74
9	FlgE	431	Hook	FlgE (VP0778) of *Vibrio parahaemolyticus*	52/58
10	FlgD	305	Rod	FlgD of *Vibrio cholerae*	49/56
11	FlgC	137	Rod	FlgC (VP0776) of *Vibrio parahaemolyticus*	74/80
12	FlgB	136	Rod	FlgB (VP0775) of *Vibrio parahaemolyticus*	59/62
13	CheR	278	Chemotaxis	CheR (VP0774) of *Vibrio parahaemolyticus*	70/76
14	CheV	313	Chemotaxis	CheV (AHA_2844) of *Aeromonas hydrophila* ATCC7966	73/81
15	FlgA	214	P-ring assembly	FlgA of *Vibrio cholerae*	39/48
16	FlgM	105	Anti-σ^28^	FlgM of *Vibrio cholerae*	44/51
17	FlgN	139	Chaperone	FlgN of *Vibrio alginolyticus*	40/45
18	FlgP	151	Flagella lipoprotein	FlgP of *Vibrio cholerae*	51/55
19	FlgO	267	Flagella lipoprotein	FlgO (VP0768) of *Vibrio parahaemolyticus*	48/55
20	FlgT	391	Flagella protein	FlgT (VP0767) of *Vibrio parahaemolyticus*	39/45

### Lateral Flagella Mutants

Several recombinant positive clones complemented E, F, and G mutants separately. Some clones were observed to complement two mutants. The complementation was studied on the basis of recovery of swarming behavior on appropriate growth plates. All complemented mutants were able to produce lateral flagella when observed by EM growing in semisolid conditions (**Figure 3**). We used the same strategy previously indicated to sequence the entire DNA region contained in the recombinant positive clones. This complete region correspond to PLESHI_07125 to PLESHI_07305 in the complete *P. shigelloides* 302-73 genome (Piqué et al., 2013).

Lateral flagella gene cluster shows 37 genes grouped in a single region (**Figure 5B**). Five typical group of genes (*lafA* to *U*; *flgB_L_* to *L_L_*; *flgA_L_*,*M_L_*,*N_L_*; *fliE_L_* to *J_L_*; and *fliM_L_* to *R_L_* plus *flhB*-*A_L_*) when compared to the most similar *A. hydrophila* AH-3 lateral flagella region were found. All the genes were found in a unique region similar to *A. hydrophila* or enteric bacteria. In contrast, in the equivalent region in *V. parahaemolyticus* is found in two separate regions (Canals et al., 2006; Merino et al., 2006). The group of genes *fliE_L_* to *J_L_* and *fliM_L_* to *R_L_* plus *flhB*-*A_L_* are adjacent in all the lateral flagella clusters described. The groups of genes have been shown to be transcribed in the same direction in *A. hydrophila* and divergently in *Vibrio*, enteric bacteria and *P. shigelloides* (Merino and Tomás, 2009). **Table 4** shows the ORFs with their predicted function based on their homology to proteins of known function. All the protein analogies that were from unknown or not well-established homology were discarded. Between the group of genes *flgB*-*L_L_* and *lafA*-*U*, there is a gene encoding for a hypothetical protein without the classical motility accessory factors domains found in *A. hydrophyla* Maf-5. However, this encoded protein showed a minimal similarity with this Maf-5, and the gene was denoted *maf-5* (Parker et al., 2014). Once the DNA fragment was completely sequenced, we used several primers derived from the DNA sequence to locate the miniTn5 in *lafA* (E), *flhA_L_* (F), and *flgE_L_* (G; **Figure 5B**).

**Table 4:** Characteristics of the *P. shigelloides* 302-73 strain lateral flagella cluster.

ORF	Protein name	Protein size	Predicted function	Homologous protein with known function	Percentage identity/similarity
1	LafU	455	Proton motor	LafU of *Aeromonas hydrophila* AH-3	41/62
2	LafT	284	Proton motor	LafT (VPA1556) of *Vibrio parahaemolyticus*	49/65
3	LafS	249	σ^28^	LafS (VPA1555) of *Vibrio parahaemolyticus*	45/64
4	LafF	158	Unknown	LafF (VPA1554) of *Vibrio parahaemolyticus*	30/52
5	LafE	404	Hook length control	LafE (VPA1553) of *Vibrio parahaemolyticus*	42/67
6	LafX	96	Chaperone	LafD (VPA1552) of *Vibrio parahaemolyticus*	21/37
7	LafC	131	Chaperone	LafC of *Aeromonas hydrophila* AH-3	52/68
8	LafB	438	Hook-associated protein 2	LafB of *Aeromonas hydrophila* AH-3	31/49
9	LafA	275	Lateral Flagellin	LafA (VPA1548) of *Vibrio parahaemolyticus*	49/65
10	Maf-5	349	Motility accessory factor	Maf-5 of *Campylobacter jejuni* subsp. *jejuni* 00-2415 Maf-5 of *Aeromonas hydrophila* AH-3	25/43 15/30
11	FlgL_L_	300	Hook-associated protein 3	FglL_L_ of *Aeromonas hydrophila* AH-3	38/43
12	FlgK_L_	467	Hook-associated protein 1	LfgK (VPA0273) of *Vibrio parahaemolyticus*	34/40
13	FlgJ_L_	328	Peptidoglycan hydrolase	LfgJ (VPA0272) of *Vibrio parahaemolyticus*	45/51
14	FlgI_L_	364	P-ring	FglI_L_ of *Aeromonas hydrophila* AH-3	66/72
15	FlgH_L_	219	L-ring	FglH_L_ of *Aeromonas hydrophila* AH-3	58/63
16	FlgG_L_	261	Rod	LfgG (VPA0269) of *Vibrio parahaemolyticus*	68/74
17	FlgF_L_	241	Rod	FglF_L_ of *Aeromonas hydrophila* AH-3	54/61
18	FlgE_L_	391	Hook	FglE_L_ of *Aeromonas hydrophila* AH-3	44/50
19	FlgD_L_	243	Rod	LfgD (VPA0266) of *Vibrio parahaemolyticus*	39/49
20	FlgC_L_	140	Rod	FglC_L_ of *Aeromonas hydrophila* AH-3	58/64
21	FlgB_L_	125	Rod	LfgB (VPA0264) of *Vibrio parahaemolyticus*	55/59
22	FlgA_L_	231	P-ring assembly	LfgA (VPA0263) of *Vibrio parahaemolyticus*	43/52
23	FlgM_L_	91	Anti-σ^28^	LfgM (VPA0262) of *Vibrio parahaemolyticus*	32/35
24	FlgN_L_	142	Chaperone	FlgN_L_ of *Aeromonas hydrophila* AH-3	46/53
25	FliJ_L_	147	Export/assembly	FliJ (VPA1532) of *Vibrio parahaemolyticus*	26/57
26	FliI_L_	443	Export ATP synthase	FliI (VPA1533) of *Vibrio parahaemolyticus*	59/66
27	FliH_L_	253	Export/assembly	FliH (VPA1534) of *Vibrio parahaemolyticus*	39/47
28	FliG_L_	337	Motor switch	FliG (VPA1535) of *Vibrio parahaemolyticus*	44/54
29	FliF_L_	569	M-ring	FliF (VPA1536) of *Vibrio parahaemolyticus*	42/49
30	FliE_L_	115	Basal body component	FliE (VPA1537) of *Vibrio parahaemolyticus*	49/56
31	FliM_L_	300	Motor switch	FliM (VPA1540) of *Vibrio parahaemolyticus*	41/52
32	FliN_L_	121	Motor switch	FliN (VPA1541) of *Vibrio parahaemolyticus*	57/64
33	FliP_L_	245	Export/assembly	FliP (VPA1542) of *Vibrio parahaemolyticus*	73/79
34	FliQ_L_	89	Export/assembly	FliQ (VPA1543) of *Vibrio parahaemolyticus*	60/69
35	FliR_L_	263	Export/assembly	FliR (VPA1544) of *Vibrio parahaemolyticus*	58/65
36	FlhB_L_	371	Export/assembly	FlhB (VPA1545) of *Vibrio parahaemolyticus*	43/49
37	FlhA_L_	701	Export/assembly	FlhA (VPA1546) of *Vibrio parahaemolyticus*	59/66

### Mutants Unable to Produce Flagella

A single recombinant positive clone was observed to complement both mutants H and I as they recover swimming and swarming in plates. The complemented mutants were able to produce polar and lateral flagella when observed by EM growing in appropriate conditions (**Figure 4**). Sequencing the entire DNA region in the recombinant positive clone showed this region to contain the group of putative biosynthetic Leg genes (**Figure 5A**) between region I and II codifying for the polar flagella. This complete region corresponds to PLESHI_03365 to PLESHI_03405 in the complete *P. shigelloides* 302-73 genome (Piqué et al., 2013).**Table 5** shows the ORFs with their predicted function based on their homology to proteins of known function.

**Table 5:** Characteristics of the *P. shigelloides* 302-73 strain gene region for legionaminic acid biosynthesis between polar flagella regions I and II.

ORF	Protein name	Protein size	Predicted function	Homologous protein with known function	Percentage identity/similarity
1	GlmU	189	Acetyltransferase	WeiJ of *Escherichia coli*	59/65
2	PtmA	254	Flagella modification protein	PtmA of *Vibrio fischeri* PtmA of *Campylobacter coli*	71/79 36/41
3	LegF	229	CMP-NeuAc synthase	NeuA of *Vibrio fischeri* Elg7 of *Escherichia coli* LegF of *Campylobacter coli*	74/80 70/73 31/37
4	PtmF	326	Oxidoreductase	(VF_0146) of *Vibrio fischeri* WeiH of *Escherichia coli* PtmF of *Campylobacter coli*	55/62 52/58 46/49
5	PtmE	352	Nucleotydil transferase	(VF_0145) of *Vibrio fischeri* Elg6 of *Escherichia coli*	63/71 61/70
6	LegH	217	*O*-acetyltransferase	NeuD (VF_0144) of *Vibrio fischeri* Elg5 of *Escherichia coli*	49/55 45/52
7	LegI	359	*N*-acetylneuraminate synthase	NeuB (VF_0143) of *Vibrio fischeri* Elg4 of *Escherichia coli*	74/79 70/74
8	LegG	382	UDP-*N*-acetylglucosamine 2-epimerase	NeuC of *Vibrio parahaemolyticus* Elg3 of *Escherichia coli* LegG of *Campylobacter jejuni*	68/72 65/78 42/47
9	LegC	382	Aminotransferase	WvaN of *Vibrio parahaemolyticus* PglE of *Vibrio parahaemolyticus*	69/75 69/75
10	LegB	395	Dehydratase	WvaM of *Vibrio parahaemolyticus* Elg1 of *Escherichia coli*	75/80 75/78

The *Campylobacter jejuni* CMP-Leg biosynthetic pathway described involves two segments: synthesis of a GDP-sugar building block and synthesis of the final CMP-nonulosonate which are linked by the *N*-acetyl transferase GlmU (Schoenhofen et al., 2009). We found all the genes encoding for the necessary two segments of the CMP-Leg biosynthetic pathway in this region besides the one encoding phosphoglucosamine mutase (PgmL) included in the first segment of the biosynthesis. Once the DNA fragment was completely sequenced, we used several primers derived from the DNA sequence to establish that the miniTn5 was located in *ptmA* (H) and *legH* (I; **Figure 5A**).

### Flagella Purification

**FIGURE 6:**
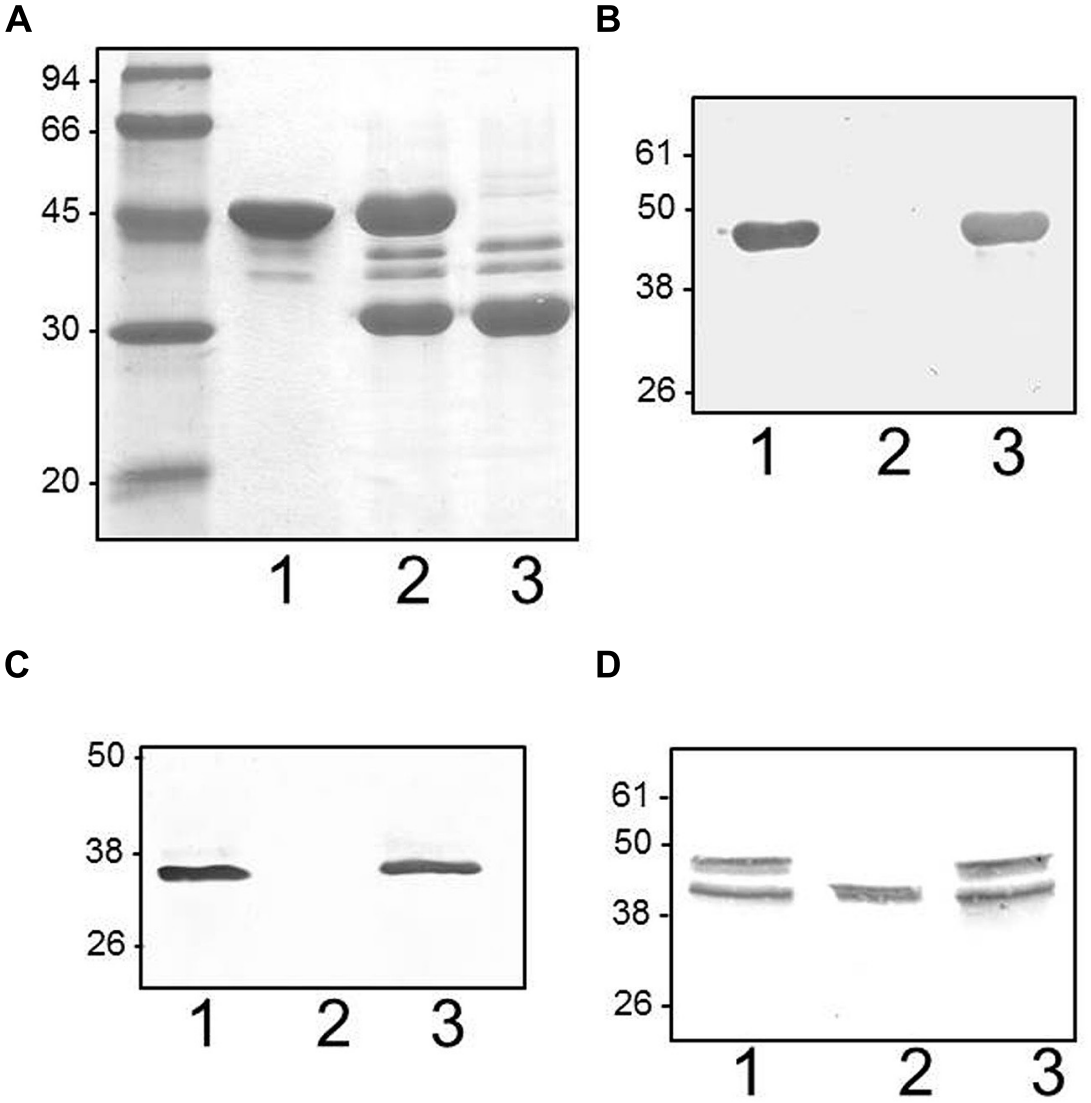
**(A)** *Plesiomonas shigelloides* 302-73 wild type strain serotype O1 purified flagella according to Section “Materials and Methods” when grown in liquid medium (1) and swarming agar plates (2). As could be observed in 2, and previously indicated in **Figure 3**, polar flagella are constitutively expressed in semisolid medium. Purified flagella from *P. shigelloides* insertional polar A mutant grown in swarming agar plates (3). **(B)**. Western blot with specific polar flagella antiserum of purified flagella from wild type (1), *P. shigelloides* insertional polar H mutant (2), and complemented mutant with COS-LEG harboring the corresponding wild type gene (3) obtained in liquid medium growth. **(C)** Western blot with specific lateral flagella antiserum of purified flagella from wild type (1), *P. shigelloides* insertional polar H mutant (2), and complemented mutant with COS-LEG harboring the corresponding wild type gene (3) obtained in swarming agar plates. **(D)** Western blot with specific polar flagella antiserum of cytoplasmic fractions obtained as described in Section “Materials and Methods” of wild type (1), *P. shigelloides* insertional polar H mutant (2), and complemented mutant with COS-LEG harboring the corresponding wild type gene (3) obtained in liquid medium growth. The low molecular weight band could correspond to the non-glycosylated form, and the upper band (not present in the mutant) to the glycosylated form.

Polar flagellins were purified from the wild type strain after grown in liquid medium and a mixture of polar and lateral flagellins after grown in swarm agar plates (**Figure 6A**). Lateral flagellin was also isolated from insertion mutant A (unable to produce constitutive polar flagella with unaltered lateral flagella).

### Intact Mass Analysis of Purified Flagellins

**FIGURE 7:**
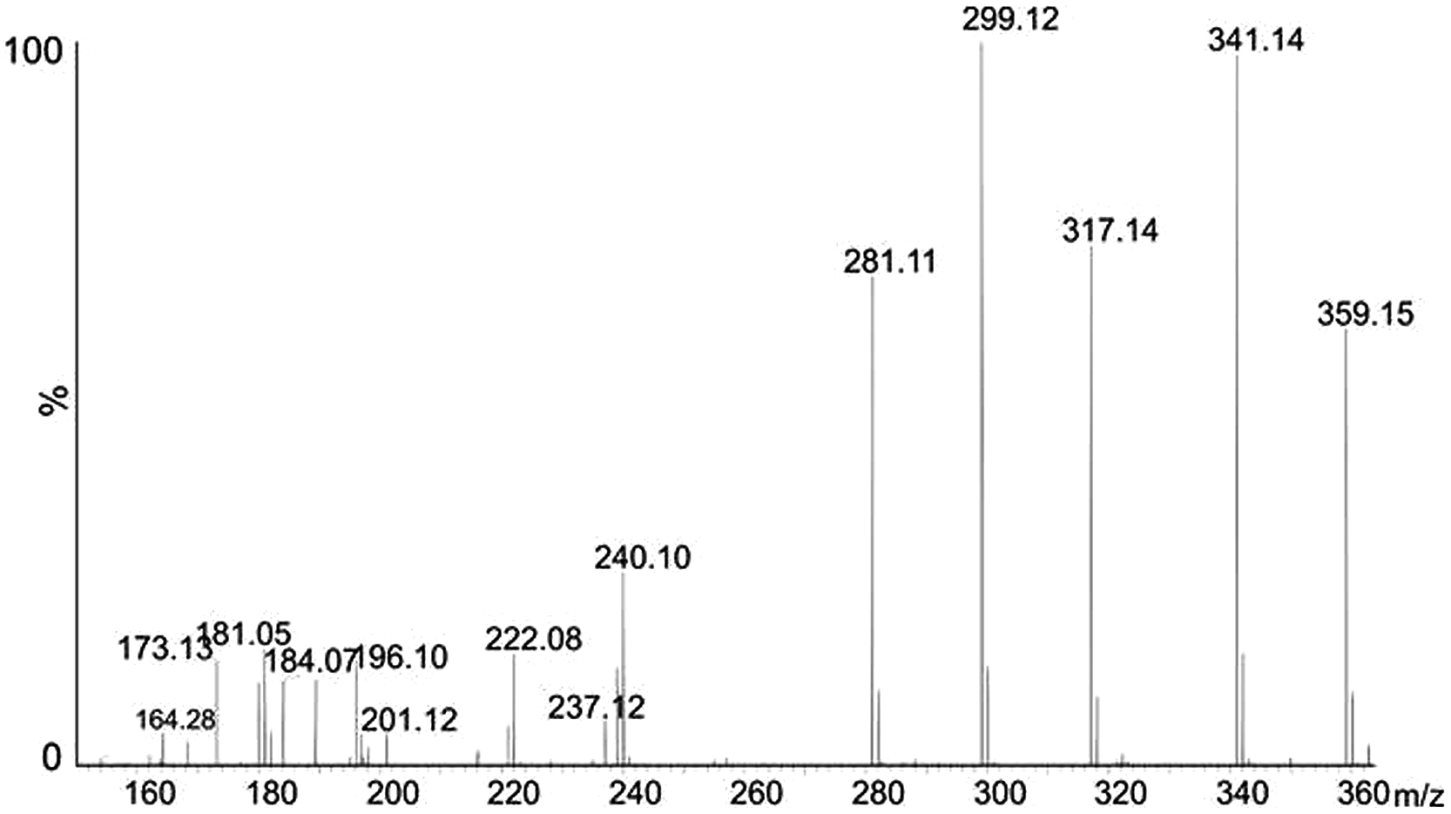
**Glycan fragmentation pattern.** Front end collision induced dissociation of polar flagellin protein, showing low *m/z* region. Fragment ions characteristic of an acetylated nonulosonic acid sugar are present.

Purified polar flagellin preparations showed a well-resolved ion envelop of multiple charged protein ions, which deconvoluted into three distinct masses at 40201, 40652, and 40931 Da. The mass of the translated gene sequence for polar flagellin was 38710 Da, giving mass excesses of 1491, 1942, and 2221 Da, respectively (data not shown). During front end CID experiments of the purified polar flagellin preparation, labile glycan related ions were observed at *m/z* 359 and 317. Using increasing cone voltages, fragmentation of this ion at *m/z* 359 was observed, as shown in **Figure 7**. The fragment ions observed at *m/z* 317, 299, 281, 222, and 181 were characteristic fragment ions of nonulosonic acids, such as pseudaminic or legionaminic acid.

From the observed mass of 316.124, the top ranked plausible elemental formula was C_13_H_21_N_2_O_6_, suggestive that this moiety is a carbohydrate. The additional glycan ion observed at *m/z* 359, gave a top ranked plausible elemental formula C_15_H_23_N_2_O_8_, suggesting this species to be a nonulosonic acid with an additional of an acetyl group. An intense fragment ion was observed at *m/z* 341, most likely a loss of water from the glycan ion observed at *m/z* 359.

The preparation containing purified polar and lateral flagellins showed a more complex elution profile when HPLC separated, with two sequentially eluting protein peaks. The area under each peak was combined separately and each showed a complex ion envelope. The ion envelope of the first eluted protein deconvoluted into two distinct masses at 39325, 40678 Da. The second eluting protein ion envelope deconvoluted to give a single protein mass at 30940. It is possible that the larger MW proteins correspond to the polar flagellin and the 30 kDa protein the lateral flagellin. The A mutant that is unable to produce polar flagella showed only this second eluting peak when grown in swarming conditions (**Figure 6A**). In each case, the measured molecular mass is greater than that of the translated gene sequence for each protein. This suggests that both polar and lateral flagellins are post-translational modified. Front end CID experiments showed almost identical profiles when compared with the polar flagellin preparation, with intense ions observed at *m/z* 359, 317. These data suggest that both polar and lateral flagellins are modified with the same nonulosonic acid sugar, with or without acetylation.

### Bottom Up Mass Spectrometry Studies of Flagellins

**FIGURE 8:**
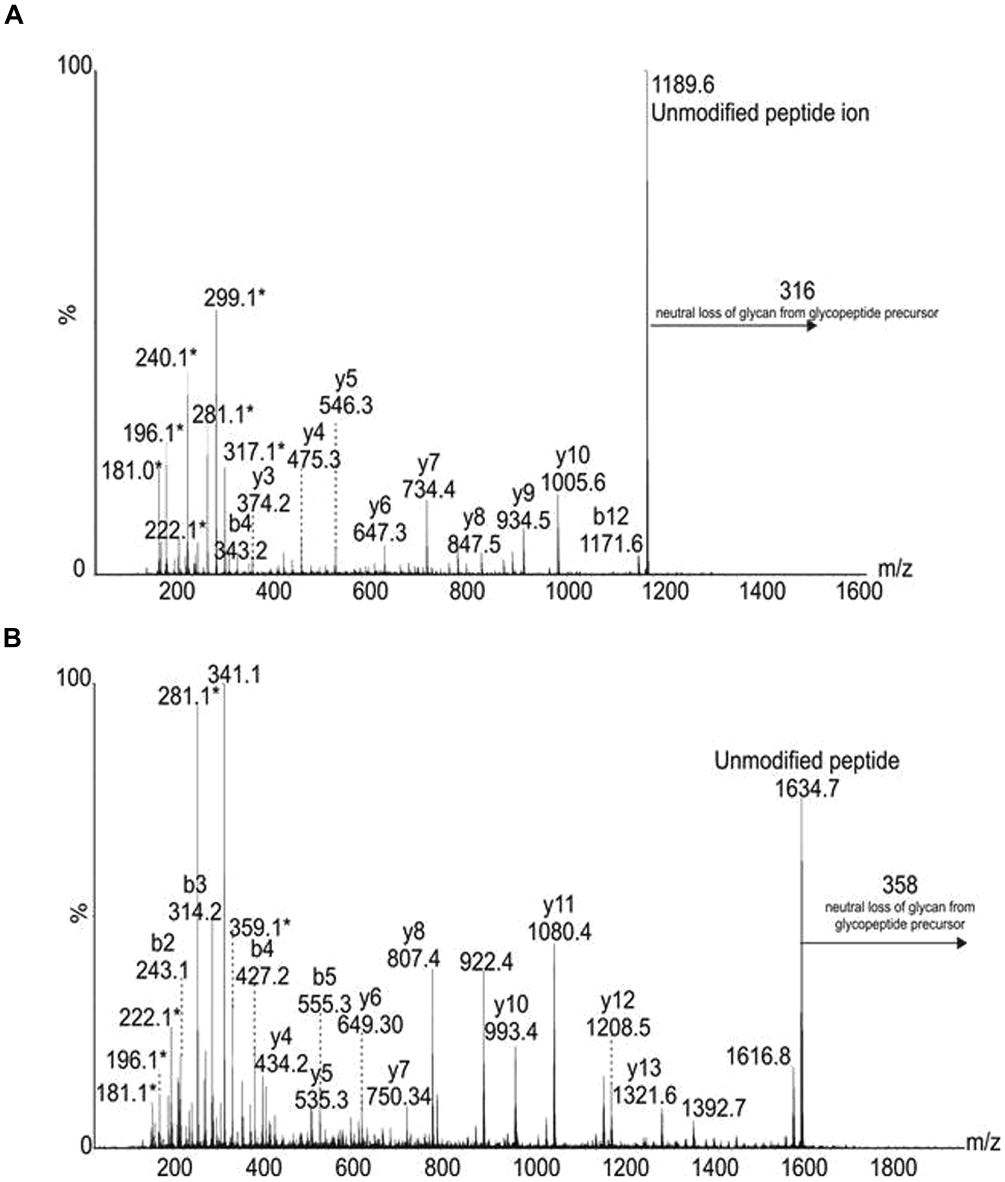
**Tandem mass spectra of flagellin glycopeptides **(A)** from polar flagellin, peptide AIASLSTAINK, modified with a 316 Da glycan.** Peptide type y and b ions are indicated, confirming the peptide sequence. In addition, glycan related fragment ions are indicated with an asterisk (^∗^), and include a glycan oxonium ion at *m/z* 317, and related ions at *m/z* 299, 281, 240, 221, and 181. **(B)** From lateral flagellin, the peptide type y and b fragment ions indicate a sequence of ELALQSANGTNTTADK. In this case the peptide is modified with a 358 Da glycan, as observed with a neutral loss of 358 from the glycopeptide precursor and the glycan oxonium ion at *m/z* 359. A loss of water is observed from this oxonium ion, in addition glycan related fragment ions are indicated with an asterisk (^∗^).

Tandem mass spectrometry studies of tryptic digests of purified polar flagellins identified a number of unmodified peptides. *De novo* sequencing of the MS/MS data showed a number of spectra that were identified as flagellin peptides and harboring mass excess of 316 Da. Also observed was an intense ion at *m/z* 317, suggestive of a glycan oxonium ion. **Figure 8A** shows the MS/MS spectrum of the polar flagellin glycopeptide AIASLSTATINK, modified with a putative 316 Da glycan. Peptide type y and b fragment ions are annotated and confirm the peptide sequence. In addition, low *m/z* fragment ions that did not correspond to peptide type y or b ions were also observed at *m/z* 317, 299, 281, 240, 221, 196, and 181. Combined with the mass excess, glycan oxonium ion and putative glycan fragment ions, the data suggest the flagellin peptides to be modified with a legionaminic acid like glycan.

The purified polar and lateral flagellins were also digested with trypsin and analyzed by tandem mass spectrometry, identifying a number of unmodified flagellin peptides. Once again, *de novo* sequencing showed several flagellin peptides from both polar and lateral flagellins to be modified with putative glycan moieties. The lateral flagellin (LafA) harbored peptides modified with glycans of 316 and 358 Da (**Figure 8B**). In some cases peptides were showed to harbor both glycans. It was not clear from the data whether two monosaccharides were modifying two separate amino acids, or whether a single disaccharide was modifying at one site.

The polar flagellin was also observed to be modified with 316 and 358 Da glycan moieties. In some cases, glycan chains comprised of multiple 358 Da glycans were observed; in other cases a single modification of 316 or 358 Da was noted. Very low levels of peptides harboring distinct glycan masses were observed, such as the peptide AIASLSTATINK, was observed to be modified with either 316 Da glycan, or a 523 or 481 Da glycan. Glycan related ions were observed in each case, with intense ions observed at *m/z* 524 and 184 or *m/z* 424 and 184. The ion at *m/z* 184 was also observed in front end CID experiments with the intact polar and lateral flagellin preparations, and gave a top ranked plausible elemental formula of C_9_H_12_O_4_, suggesting that it is a related nonulosonic acid type sugar. The low abundance of these glycopeptides made any further analyses challenging.

### Legionaminic Acid Biosynthetic Mutants

The insertional mutants in *ptmA* (H) and *legH* (I) were unable to produce polar or lateral flagella under induced conditions, as shown by TEM or by immunodetection (**Figure 6B**) or lateral flagellins (**Figure 6C**) in purified flagella. The introduction of the *P. shigelloides* wild type genes was observed to recover the production of polar and lateral flagella in the mutants. This was demonstrated using immunodections, as shown in **Figures 6B,C**. These data prompted us to examine the production of the polar flagellin in the mutants by immunodetection. Western blot analysis shows presence of polar flagellin the cytoplasmic subcellular fraction. Interestingly, only a single protein band was observed, with a lower than expected molecular weight (**Figure 6D**). Wild type flagellin typically migrates as two distainct bands, both detectable by Western blot. We speculate that the single, lower molecular weight species is a non-glycosylated form of flagellin. The complemented mutants showed the same cytoplasmic polar flagellin molecular weight bands as observed with wild type strain. Similarly, where lateral flagellin was detected in the cytoplasmic fraction, it was observed at a lower molecular weight, likely the non-modified form of the protein. Then, the lack of polar and lateral flagella formation observed in the mutants is not by the lack of flagellin protein or the master regulator transcription.

In order to prove at the genomic level that mutations in the CMP-Leg biosynthetic pathway were responsible for the phenotypic traits shown by insertional mutants H and I, two in-frame *pgmL* and *legF* deletion mutants were generated, 302Δ*pgmL* and 302Δ*legF*, respectively. Our genomic studies indicates that all the genes of the Leg pathway are included in the cluster between polar region I and II, with the exception of the PgmL ortholog which is found in another region of the chromosome [703.5 peg 1785 (Piqué et al., 2013)]. PgmL or GlmM, phosphoglucosamine mutase, is involved in the first step to produce GDP-GlcNAc. LegF, CMP-legionaminic acid synthase is the final enzyme of the second step to produce CMP-Leg. Using TEM, neither mutant was observed to produce polar or lateral flagella under induced conditions. Both show the same phenotypic traits as insertional mutants H and I. When mutants 302Δ*pgmL* and 302Δ*legF* were complemented with their single corresponding wild type gene (pBAD33-*pgmL* and pBAD33-*legF*, respectively) under inducing conditions (plus arabinose) all the wild type phenotypic traits (production of polar and lateral flagella or swimming and swarming motilities) were fully recovered. Control plasmid pBAD33 alone under inducing conditions (plus arabinose) was unable to do it.

### Lateral Flagella and Leg *O*-Flagella Glycosylation Gene Distribution on *P. shigelloides*

**FIGURE 9:**
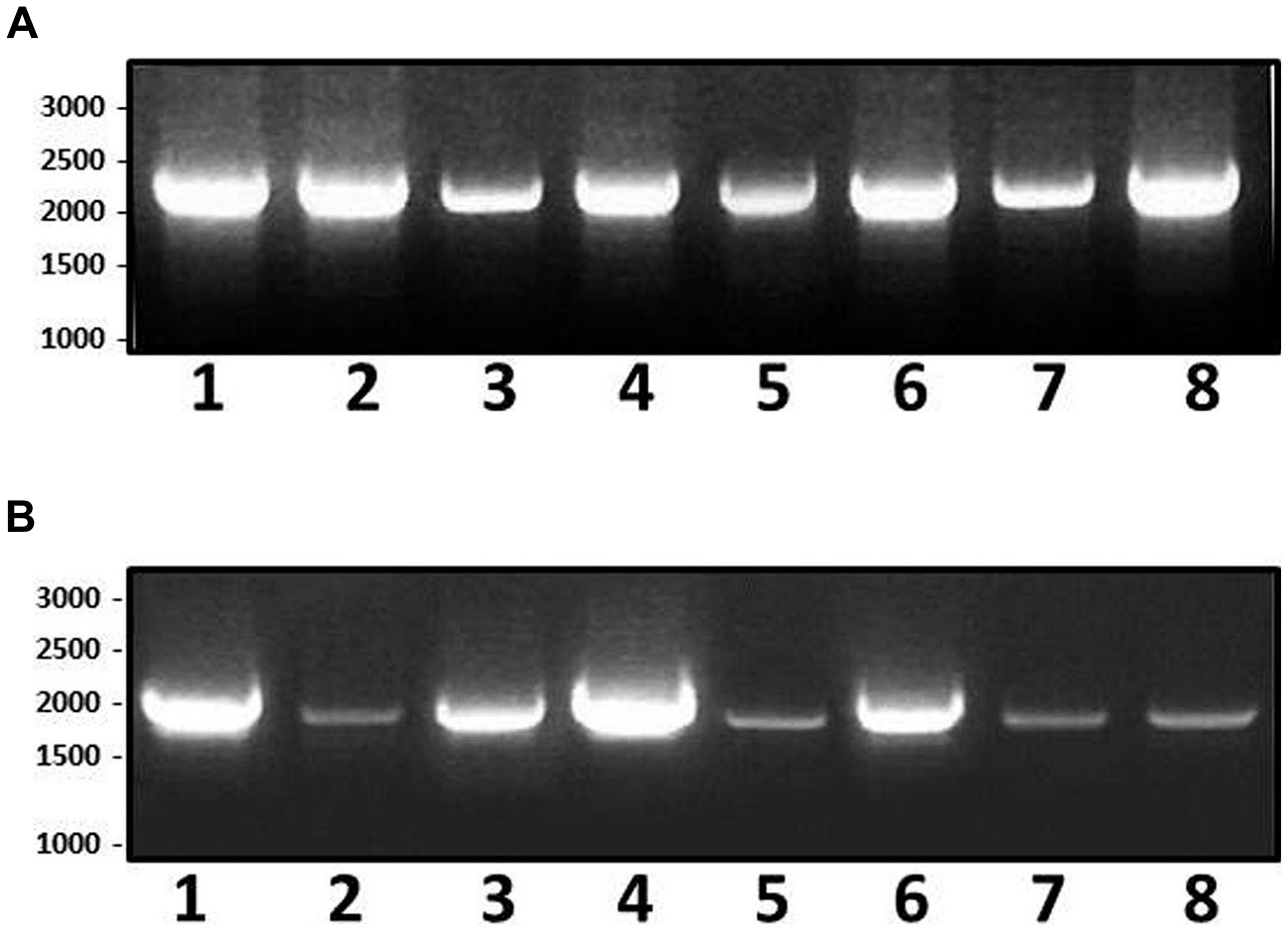
**Polymerase chain reaction (PCR) amplified bands from genomic *P. shigelloides* DNAs of strains: 302-73, serotype O1 (1); 306-73, serotype O2 (2); 307-73, serotype O3 (3); 343-73, serotype O17 (4), PCM2647, serotype O54 (5), C1, non-serotyped from Spain (6); P12, non-serotyped from Spain (7); and lt645, non-serotyped from Brazil (8); using the set of oligonucleotides for lateral flagella cluster (**A**, 2160 bp DNA fragment) and Leg biosynthesis genes (**B**, 1985 bp DNA fragment)**.

In order to test if the presence of lateral flagella and Leg *O*-flagella glycosylation genes is a specific feature for the strain studied, the 12 previously mentioned *P. shigelloides* strains used for PCR studies were eight strains representing five different serotypes (O1, O2, O3, O17, and O54) plus 4 non-serotyped strains described in Material and Methods. Initially, genomic DNA from 302-73 strain was used as template for PCR amplification with two sets of oligonucleotides: 5′-ATCGCGTCTGAAAGGCTAC-3′ and 5′-CTGCGCCATAGAACTACCC-3′ which amplified a 2160 bp DNA fragment from lateral flagella cluster (partial *lafA* and complete *maf-5*); and another oligonucleotide set (5′-CGGGTTAAAGCTATCCCATC-3′ and 5′-CCAATGACAGCTGAATCTCC-3′) amplified a 1985 bp DNA fragment from Leg biosynthesis genes (partial *legH* and complete *legI*). DNA fragments of the same size (2160 and 1985 bp, respectively) were PCR amplified for all the genomic DNAs from the strains studied, as shown by the results shown in **Figure 9**. DNA sequence of the amplified fragments confirmed the presence of the lateral and Leg biosynthetic genes. In addition, in all the amplified *maf-5* and *legI* fragments the presence of a sequence coding for the N-terminal amino acid residues of *lafA* and *legH* genes, respectively, were found adjacent to *maf-5* or *legI*, suggesting that in the analyzed strains the genomic location is the same as that found in *P. shigelloides* wild type strain 302-73 (**Figure 5**).

## Discussion

Motility is an essential mechanism in adaptation to different environments for free living bacteria. Bacteria showed three flagella types classified according to their location on a cell: peritrichous, polar, and lateral. It has been reported dual flagella systems in some polar flagellated bacteria when grow in viscous environments or surfaces. This fact allows bacteria to swarm on solid or semisolid media by a mixed flagellation (polar and lateral flagella). *P. shigelloides* has been observed to express mixed flagellation (Inoue et al., 1991).

Two *P. shigelloides* 302-73 different gene clusters were described, one exclusively involved in lateral flagella biosynthesis, and a second containing the polar flagella genes distributed in two regions spaced by putative glycosylation genes. It is characteristic of the bacteria with dual flagella systems to separate both in different gene clusters (McCarter, 2001; Canals et al., 2006; Merino et al., 2006; Merino and Tomás, 2009). Of note, *P. shigelloides* is the first *Enterobacteriaceae* with lateral flagella production as shown herein.

*Plesiomonas shigelloides* lateral gene cluster is nearly identical to the lateral gene cluster of *A. hydrophila* according to the gene grouping and transcription direction, with the exception of the group of genes *fliM_L_* to *R_L_* plus *flhB*-*A_L_* which are transcribed in opposite direction (Canals et al., 2006). However, no *lafK* ortholog could be detected in *P. shigelloides* lateral gene cluster. This gene has been reported in all the lateral gene clusters, including the non-functional in the *Enterobacteriaceae* (Canals et al., 2006; Merino and Tomás, 2009). A non-functional Flag-2 flagella cluster with large similarity to *V. parahaemolyticus* lateral flagella system, was found in different *E. coli* enteroaggregative or *Yersinia pestis* or *pseudotuberculosis* strains (Ren et al., 2004). However, as we proved, *P. shigelloides* lateral gene cluster is fully functional.

The transcriptional hierarchy of *V. parahaemolyticus* lateral flagella is one of the *Gammaproteobacteria* model. LafK (σ^54^-associated transcriptional activator) is the master regulon in this model, controlling Class II lateral flagella genes transcription. Class II genes contains the σ^28^ factor (*fliA*_L_) which is involved in transcription of Class III lateral flagella genes (Stewart and McCarter, 2003). In *V. parahaemolyticus* the absence of polar flagellum induces the expression of lateral flagella in liquid medium, and LafK is able to compensate the lack of FlaK (σ^54^-associated polar transcriptional activator) and activate polar flagellum class promoters. *A. hydrophila* lateral flagella transcriptional hierarchy represents the second *Gammaproteobacteria* model. Class I gene transcription in *A. hydrophila* lateral flagella is σ^70^-dependent as LafK in contrast to describe in *V. parahaemolyticus* (Stewart and McCarter, 2003). It is important to point out that *A. hydrophila* lateral flagella genes are transcribed in liquid and solid or semisolid media, and unlike *V. parahaemolitycus* the genes are not induced by mutation of polar flagellum genes. The transcription hierarchy of *A. hydrophila* lateral flagella is complex because LafK is not strictly their master lateral flagella regulator, and many clusters of genes are LafK independently transcribed (Wilhelms et al., 2013). *A. hydrophila* LafK protein is unable to not compensate the lack of FlrA, which is the polar-flagellum regulator (σ^54^-associated transcriptional activator for polar flagellum), a situation that happens in *V. parahaemolyticus* (Wilhelms et al., 2013). This point is in agreement with *A. hydrophila* FlrA mutation not affect lateral flagella besides that abolishes polar flagellum formation in liquid and on solid surfaces (Wilhelms et al., 2013).

The *P. shigelloides* polar flagella gene regions show greater similarity to those reported in *Vibrio* or *Aeromonas* than the regions in *Enterobacteriaceae* [e.g., *E. coli* or *S. typhimurium* (Chilcott and Hughes, 2000)]. Bacteria with peritrichous flagella, such as *E. coli* and *Salmonella*, showed three hierarchy levels. The σ^70^ is required for transcription of class I and II genes, and class I promoter responds to different regulatory factors and transcribes the FlhDC master activator, which allowed the class II σ^70^-dependent promoter expression. At the top of the *Vibrio* sp. or *A. hydrophyla* polar flagella hierarchy is σ^54^-associated transcriptional activator (FlrA, named FleQ in *Pseudomonas aeruginosa*) which activates class II genes σ^54^-dependent promoters. Class II promoters encode a two component signal-transducing system (*Vibrio* sp. or *A. hydrophyla* FlrBC and FleSR in *P. aeruginosa*) whose regulator (FlrC/FleR) activates class III genes σ^54^-dependent promoters.

In the *P. shigelloides* polar flagella region I only *flrA* and *C* orthologs were observed. *P. shigelloides* FlrA shows the characteristic three domains (FleO, σ^54^ -interaction domain and family regulatory protein Fis) like in *Vibrio* sp. or *A. hydrophila* (Kim and McCarter, 2004; Wilhelms et al., 2011). Class II promoters encode a two component signal-transducing system (FlrBC of *Vibrio* sp. or *A. hydrophila* and FleSR in *P. aeruginosa*) whose regulator (FlrC/FleR) activates class III σ^54^-dependent promoters. However, when analysis of *P. shigelloides* FlrC encoded protein, revealed the corresponding domains for FlrB and C. Thus, *P. shigelloides* FlrC contains two domains of *Vibrio* sp. or *A. hydrophila* FlrB (PAS domain and His Kinase A) as well as two domains of *Vibrio* sp. or *A. hydrophila* FrlC (σ^54^-interaction domain and family regulatory protein Fis). We suggest that *P. shigelloides* FlrC could be able to activate class III genes σ^54^-dependent promoters as observed in *Vibrio* sp. or *A. hydrophila*. No FlrB ortholog was observed in the *P. shigelloides* 302-73 genome (Piqué et al., 2013). It could be suggested that in *P. shigelloides*, FlrB and C functions are developed by a single bifunctional protein encoded by the single *flrC* as it happens for some LPS-core biosynthetic genes (Jiménez et al., 2009). Taken together, the data presented hererin, no *lafK* or separate *flrB* in *P. shigelloides*, indicate that their lateral and polar flagella transcriptional hierarchy represents a different *Gammaproteobacteria* model that requires further study.

Among this large *P. shigelloides* polar flagella gene cluster, genes were identified between the two polar flagella regions, the presence of genes putatively linked to glycosylation. These genes were not found in other *Enterobacteriaceae* studied. *O*-glycosylation could be performed by a mechanism dependent or not of an oligosaccharyltransferase (OTase; Kim and McCarter, 2004; Iwashkiw et al., 2013). The *O*-glycosylation frequently affects protein stability, flagella filament assembly, bacterial adhesion, biofilm formation, and virulence in general as has been described in several bacteria (Lindenthal and Elsinghorst, 1999; Logan, 2006; Faridmoayer et al., 2008; Egge-Jacobsen et al., 2011; Iwashkiw et al., 2013; Lithgow et al., 2014). The predominant *O*-glycans linked to flagellins are mainly derivatives of pseudaminic acid (PseAc, where Ac represents an acetamido group) and in a minor extent an acetamidino form of legionaminic acid (LegAm, where Am represents acetamidino; Merino et al., 2014). Both are nine-carbon sugars related to sialic acid. The flagellin glycosylation pathways in both cases have been elucidated, including the Pse pathway of *Helicobacter pylori* and *C. jejuni* (Fox, 2002), the Leg pathway of *C. jejuni* (Schoenhofen et al., 2009). Until today the Leg flagella glycosylation has been restricted to *C. jejuni* or *coli*. The CMP-legionaminic acid biosynthetic pathway in *C. jejuni* involves two steps: synthesis of a GDP-GlcNAc and synthesis of the final CMP-Leg (Schoenhofen et al., 2009). The insertional mutants obtained *ptmA* (H) and *legH* (I), represent key eznymes in the first and second steps of the CMP-Leg biosynthesis, confirming the observation data that both mutants are unable to produce polar or lateral flagella. Furthermore, the in frame mutants obtained in *pgmL* and *legF*, one enzyme of the first step and the last enzyme of the second step of the CMP-Leg biosynthesis, respectively, clearly confirmed the legionaminic acid polar and lateral glycosylation as both mutants are unable to produce polar or lateral flagella as it happens with the insertional mutants.

Mass spectrometry studies show that both flagella in *P. shigelloides* strain 302-73 are glycosylated by a derivative of Leg, and is also indicated by the presence of Leg biosynthetic pathway genes nearby the polar flagella gene regions. It is the first *Enterobacteriaceae* reported to harbor *O*-glycosylation modification on both polar and lateral flagella. Moreover, it is also the first bacteria reported to express a lateral flagella glysosylated by Leg. We also demonstrated that flagella *O*-glycosylation is essential for bacterial flagella formation, either polar or lateral. However, the flagella *O*-glycosylation is not determinant for cytoplasmic flagellin production as can be observed by immunodetection studies.

The *P. shigelloides* homologous recombination rates are extremely high (Salerno et al., 2007), like naturally transformable species as *Streptococcus pneumonia*e. In the rest of *Enterobacteriaceae* the recombination rate is much lower. The high recombination observed in this bacterium could offer a reason for *P. shigelloides* variety of LPS-core structures (Salerno et al., 2007). The PCR experiments using several *P. shigelloides* strains and lateral flagella or Leg pathway genes, with the motility and EM studies, demonstrated that presence of lateral flagella and Leg *O*-flagella glycosylation is a widely spread feature, not a strain specific observation. Furthermore, the maintenance of these genes among the different strains besides the recombination rate observed for *P. shigelloides*, indicates the importance of glycosylated polar and lateral flagella production for this bacterium.

## Conflict of Interest Statement

The authors declare that the research was conducted in the absence of any commercial or financial relationships that could be construed as a potential conflict of interest.
